# Change in Ocular Surface Staining during Eyelid Warming Is Related to Tear Cytokine Levels

**DOI:** 10.1155/2022/5103231

**Published:** 2022-08-03

**Authors:** Erlend C. S. Landsend, Jonatan Olafsson, Xiaoran Lai, Hans C. D. Aass, Tor P. Utheim

**Affiliations:** ^1^Department of Ophthalmology, Oslo University Hospital, P.O. Box 4950 Nydalen, Oslo 0424, Norway; ^2^Institute of Clinical Medicine, Faculty of Medicine, University of Oslo, P.O. Box 1171 Blindern, Oslo 0318, Norway; ^3^Department of Medical Biochemistry, Oslo University Hospital, P.O. Box 4950 Nydalen, Oslo 0424, Norway; ^4^The Norwegian Dry Eye Clinic, Ole Vigs Gate 32E, Oslo 0366, Norway; ^5^Oslo Centre for Biostatistics and Epidemiology, Faculty of Medicine, University of Oslo, P.O. Box 1122 Blindern, Oslo 0317, Norway; ^6^National Centre for Optics, Vision and Eye Care, Department of Optometry, Radiography and Lighting, Faculty of Health and Social Sciences, University of Southeast Norway, P.O. Box 235, Kongsberg 3603, Norway; ^7^Schepens Eye Research Institute, Massachusetts Eye and Ear, Department of Ophthalmology, Harvard Medical School, 20 Staniford St., Boston 02114, MA, USA; ^8^Department of Ophthalmology, Sørlandet Hospital Arendal, P.O. Box 416, Kristiansand 4604, Norway; ^9^Department of Ophthalmology, Stavanger University Hospital, P.O. Box 8100, Stavanger 4068, Norway; ^10^Department of Research and Development, Oslo Metropolitan University, P.O. Box 4, Oslo 0130, Norway; ^11^Department of Clinical Medicine, Faculty of Medicine, University of Bergen, P.O. Box 7800, Bergen 5020, Norway; ^12^Department of Quality and Health Technology, The Faculty of Health Sciences, University of Stavanger, P.O. Box 8600, Stavanger 4036, Norway; ^13^Department of Ophthalmology, Vestfold Hospital Trust, P.O. Box 2168, Tønsberg 3103, Norway

## Abstract

**Purpose:**

To investigate the changes in the tear cytokine profile of patients with meibomian gland dysfunction (MGD) treated with eyelid warming and to correlate these changes with clinical parameters for dry eye disease (DED).

**Methods:**

Seventy patients with MGD were included and treated with the warming of eyelids. Of these, 61 still used the treatment three months after baseline, while 48 completed the whole treatment period of six months. The concentrations of 39 cytokines in the tear fluid were measured at baseline and after three and six months of treatment. All participants were examined with tests for DED, including tear film break-up time (TBUT), ocular surface staining (OSS), and the self-reporting Ocular Surface Disease Index (OSDI). Changes in cytokine concentrations were assessed from baseline to three months, from three to six months, and from baseline to six months. Correlation analyses were performed between changes in the cytokine concentrations and changes in TBUT, OSS, and OSDI during the same time intervals.

**Results:**

No significant changes were found in the concentrations of the 39 cytokines during any of the three treatment intervals. However, several correlations were detected between changes in the level of cytokines and OSS from baseline to three months of treatment. Decreasing concentrations of granulocyte chemotactic protein 2 (GCP-2/CXCL6, mean effect 2.36, *p*=0.042), interleukin 10 (IL-10, mean effect 1.04, *p*=0.045), and IL-16 (mean effect 1.36, *p*=0.035) were associated with decreasing OSS. Decreasing concentrations of granulocyte macrophage colony-stimulating factor (GM-CSF, mean effect −2.98, *p*=0.024), IL-8 (IL-8/CXCL8, mean effect −1.35, *p*=0.026), and macrophage migration inhibitory factor (MIF, mean effect −2.44, *p*=0.033) were related to increasing OSS.

**Conclusions:**

Warming of eyelids did not change the concentration of cytokines in the tear fluid of patients with MGD significantly. However, alterations in the level of several cytokines were associated with changes in the OSS. This finding indicates a close connection between tear cytokines and OSS in MGD patients treated with eyelid warming.

## 1. Introduction

Meibomian gland dysfunction (MGD) is an abnormality of sebaceous glands in the eyelids named meibomian glands. The glands are located in the tarsal plate of the eyelids and secrete lipids that cover and protect the aqueous layer of the tear film [1]. MGD is the major cause of dry eye disease (DED) [2]. It is characterized by a chronic, diffuse abnormality of the meibomian glands, which may lead to altered production or quality of the tear film lipids, or both [3]. Impaired function of the tear film lipid layer could result in increased evaporation of the aqueous layer, elevated tear film osmolarity, inflammation, and damage to the ocular surface epithelium.

The concentration of inflammatory cytokines in the tear fluid is elevated in DED [4]. Increased tear cytokine levels have also been found in patients with DED related to MGD [5]. Higher concentrations of these inflammatory cytokines are associated with more severe DED [4, 5]. Inflammation can lead to damage and death of goblet cells and epithelial cells at the ocular surface [6]. Inflammation may also decrease the stability of the corneal epithelial cells by degrading epithelial barriers. Hence, direct damage to the ocular surface epithelium caused by increased evaporation in MGD could be enhanced by inflammation, in a vicious circle.

Warming and subsequent massage of the eyelids is a fundamental treatment for MGD [7]. Eyelid warming is expected to improve secretion from the meibomian glands by melting dysfunctional lipids in the glands [7]. The following massage may facilitate gland secretion. Improved excretion of lipids from the meibomian glands could stabilize the tear film, reduce evaporation, and prevent damage of the ocular surface. We have previously investigated the efficacy of eyelid warming with heated steam compared with dry heat [8]. In the current study, we hypothesize that eyelid warming changes the concentrations of cytokines in the tear fluid of patients with MGD. Furthermore, we hypothesize that changes in cytokine concentrations correlate with clinical parameters of DED. Thus, our aim of this study was to compare the level of tear cytokines before and after eyelid warming with either a steam-based or dry heat technique and explore associations between cytokine levels and clinical variables during treatment.

## 2. Materials and Methods

### 2.1. Study Design and Participants

The study was part of an open-label, randomized comparative trial [8] (Clinicaltrials.gov ID: NCT03318874). The trial compared eyelid warming with either Therapearl eye mask (Bausch and Lomb Inc., New York, USA) or Blephasteam (Spectrum Thea Pharmaceuticals LTD, Macclesfield, UK) for the treatment of MGD. Participants were recruited from the Norwegian Dry Eye Clinic (Oslo, Norway) or Lavista Optometry Clinic (Lillestrøm, Norway). They were included in the study if they were above 18 years and diagnosed with MGD [9]. The diagnostic criteria for MGD in our study were Ocular Surface Disease Index score >12 [10]; tear film break-up time (TBUT) <10 seconds in at least one eye [11, 12]; the Schirmer test without anesthesia >5 mm in at least one eye [11]; and meibomian expressibility or quality score >1 by age ≤20 years or ≥1 by age >20 years [13]. Participants were excluded if diagnosed with glaucoma, ocular allergy, or autoimmune disease, and if they wore contact lenses during the study; had current punctal plugging; were pregnant or lactating; were candidates for topical anti-inflammatory therapy; or had cicatricial MGD.

The participants were randomized into treatment with either Blephasteam or Therapearl [8]. They were examined at the start of treatment (baseline), and after three and six months. However, the last eight participants to be included were only examined at three months after the start of intervention due to logistical limitations. The participants registered their compliance to treatment in a diary.

The study was approved by the Norwegian Regional Committees for Medical and Health Research Ethics (reference: 2014/1983) and conducted in accordance with the Declaration of Helsinki. All participants gave written consent after receiving oral and written information about the study.

### 2.2. Interventions

Blephasteam is designed as swimming goggles, which are sealed to the skin upon wearing. It delivers heated steam to the eyelids. The device was preheated and wetted cotton rings were placed inside the goggle chambers. The participants wore the goggles for 10 minutes once daily.

Therapearl eye mask delivers dry heat to the eyelids. In accordance with the user guide, the mask was heated in a microwave oven for 10–15 seconds and placed upon closed eyes. The participants used the eye mask for 10–15 minutes once daily.

All participants were taught to massage the eyelids for 10–15 seconds after each treatment with Blephasteam or Therapearl. They were provided an artificial tear substitute (Hylo-Comod, Ursapharm, Saarbrücken, Germany) to apply as needed to relieve acute symptoms of DED.

### 2.3. Evaluation of the Ocular Surface

All participants completed the Ocular Surface Disease Index questionnaire at each visit [10]. This questionnaire evaluates symptoms from DED. A standardized clinical examination of the ocular surface was performed. The following parameters were assessed: (1) tear production with the Schirmer test without anesthesia [11]; (2) TBUT [11, 12]; (3) ocular surface staining (OSS) [14]; and (4) meibomian gland expressibility and meibum quality [13]. One investigator (JO) completed all examinations under similar conditions for all participants.

### 2.4. Collection and Analysis of Tear Fluid

Tear fluid was collected with Schirmer Tear Test Strips (Haag-Streit UK, Essex, UK) and without topical anesthesia. The strip was stored in a cuvette containing 500 microliters of phosphate-buffered saline. The cuvette was thereafter placed in a deep freezer at −80°C until analysis.

Total protein concentrations in the Schirmer strip suspensions were determined using the Pierce BCA Protein Assay Kit (Thermo Scientific, Rockford, IL, USA) and expressed as mg/ml. Cytokine concentrations in the suspensions were measured with a procedure described previously (modified) [15]. Phosphate-buffered saline was added to a final concentration of 0.5% prior to analysis. The multiplex assay was optimized for the detection of low concentrations. In the current study, the screening kit allowed the analysis of 40 different cytokines (Table 1; Bio-Plex Pro Human Cytokine 40-plex Assay, Cat. No. 171AK99MR2, Bio-Rad, Hercules, CA, US). Samples from the individual patient were analyzed on the same microplate. All Schirmer tests were analyzed, independent of the amount of wetting of tear fluid. The cytokine levels were adjusted with total protein concentration and expressed as (pg of cytokine)/(mg of total protein).

### 2.5. Quantitative and Statistical Analyses

Cytokine concentrations from the right eye were used in the statistical analysis. Prior to analysis, all cytokine concentrations were first scaled by each patient's Schirmer test results and the resulting data underwent log2-transformation. Changes in cytokine levels between the eye examinations were assessed in the Blephasteam group and the Therapearl group separately, and in one group merging all participants in the study. Hence, calculations were performed in three different groups. The differences in cytokine concentrations were tested with the paired *t*-test in R, version 4.0.3. (The R Foundation for Statistical Computing, Welthandelsplatz 1, 1020 Vienna, Austria). The Benjamin–Hochberg procedure was employed to correct for multiple testing. Correlation between changes in cytokine levels in the right eye and changes in clinical parameters in this eye was tested with a generalized linear regression model and only in the merged group of all participants in the study. *P* values <0.05 were considered statistically significant.

## 3. Results

In total, 70 participants were included in the study. Of these, 37 were treated with Blephasteam and 33 with Therapearl. Tear cytokine concentrations at baseline of the study were available from 68 of the 70 participants. The mean age of these 68 participants was 54.7 (95% CI 50.5–58.9) years. Three months after baseline, 61 (87.1%) of the subjects were still using the eyelid warming. Cytokine levels after three months of treatment were accessible from 60 of these 61. Six months after baseline, number of subjects using eyelid warming had dropped to 48 (68.6%), and cytokine levels after six months were available from 47 of these.

### 3.1. Changes in Cytokine Levels after Treatment

No statistically significant changes in cytokine concentrations were found in any of the three groups, neither from baseline to three months nor from baseline to six months. One of the cytokines (ENA-78/CXCL5) was excluded from the analyses since its value was missing from most of the samples.

**Figure 1:**
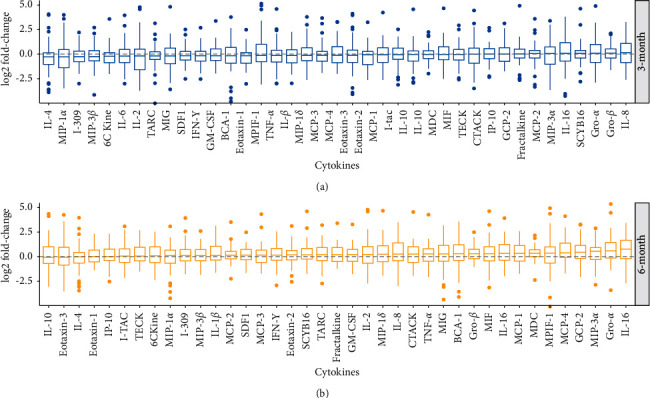
Changes in cytokine concentrations from baseline to three months of treatment (a) and from baseline to six months of treatment (b). Changes are displayed from left to right in order of cytokine with most reduced median concentration to most increased. The *y*-axis corresponds to log2 fold changes in concentrations.

Figure 1 shows a graphical presentation of changes in cytokine levels in the whole group of participants from baseline to three months, and from baseline to six months. We assessed the number of cytokines with decreasing or increasing concentration, without regard to statistical significance. The concentration of most cytokines decreased from baseline to three months, although, from baseline to six months, the concentration of most cytokines increased. Accordingly, it appeared to be a trend towards lower concentrations in the whole group of cytokines after three months of treatment. Moreover, the trend seemed to go in the direction of increasing cytokine concentrations after six months of treatment.

### 3.2. Correlation between Cytokine Levels and Dry Eye Disease Findings

We examined associations between changes in cytokine levels and changes in clinical parameters from baseline to three months. Correlations were not calculated from baseline to six months due to insufficient number of patients completing the treatment until six months. The Schirmer test results were excluded from the analysis as they were already used to adjust the cytokine concentrations. The correlations were calculated in the group of all participants.

Statistically significant correlations were found between changes in OSS and the concentrations of six cytokines from baseline to three months. We also detected a significant correlation between change in the level of one cytokine and TBUT in the same time period. The results are presented in Table 2. A decrease in the level of granulocyte chemotactic protein 2 (GCP-2), interleukin 10 (IL-10), and IL-16 was associated with the reduced severity of OSS. On the other hand, a decrease in granulocyte macrophage colony-stimulating factor (GM-CSF), IL-8, and macrophage migration inhibitory factor (MIF) was related to increased severity of OSS. Furthermore, reduction in the concentration of monocyte chemoattractant protein 3 (MCP-3) correlated with longer TBUT.

Our results suggest that the concentration of GM-CSF, IL-10, MCP-3, and MIF decreased from baseline to three-month follow-up. However, the level of GCP-2, IL-8, and IL-16 seemed to increase in this treatment period. We compared these results with data from the correlation analysis. The possible decrease in the level of GM-CSF and MIF would be associated with increasing OSS. However, a reduction in IL-10 would be related to a lower OSS score. A rise in the level of GCP-2 and IL-16 is connected to a higher OSS score. On the other hand, the apparent increase in IL-8 would be related to reduction in OSS. Eventually, the suggested decrease in MCP-3 will be associated with longer TBUT.

## 4. Discussion

Our study shows no significant changes in the level of inflammatory tear cytokines after eyelid warming in patients with MGD. However, several correlations were found between alterations in cytokine concentrations and OSS after three months of treatment.

Inflammatory cytokines are present in the tear fluid of healthy individuals [16]. These cytokines have both pro- and anti-inflammatory qualities [17], and are taking part in the defense system of the ocular surface [16]. The concentration of cytokines in the tear fluid is elevated in various inflammatory conditions at the ocular surface [18, 19], including DED [4, 20]. Accordingly, patients with MGD and evaporative DED show increased levels of inflammatory tear cytokines [5]. The levels also correlate with clinical parameters for DED. This corresponds with the recognition of inflammation as a central process in the pathogenesis of DED [6, 21]. The cytokines in our test panel have a wide range of actions in the immune system. It could therefore be assumed that a specific medical intervention would influence these differently. Eyelid warming potentially impacts several mechanisms at the ocular surface, and various changes in cytokine levels could be predicted.

We investigated the concentration of 39 cytokines in the tear fluid of patients with MGD treated with eyelid warming. The concentrations were not significantly changed in any of the treatment groups or in the merged group of all participants, neither after three nor six months of treatment. This finding may indicate that eyelid warming is insufficient to alter inflammation at the ocular surface in MGD. On the other side, the cytokine levels could have been relatively normal at the start of treatment. Comparison with a control group at baseline would have been useful to investigate this relation. Patients who were candidates for topical anti-inflammatory therapy were excluded from our study. Hence, our group of patients had clinically less severe inflammation and presumably lower levels of inflammatory cytokines than the excluded patients. A study exploring the effect of eyelid warming devices on the cytokine profile in patients with MGD and severe inflammation would have been interesting. Such a study, however, could be challenging to perform as these patients are candidates for both anti-inflammatory treatment and eyelid warming. Thus, it would be difficult to discern how these two therapies influence the cytokine profile.

The possible variation in cytokine levels in response to treatment (Figure 1) may reflect a change in the relationship between the cytokines rather than definite reduction in concentrations, which could have been expected. Still, our findings imply that the level of most of the cytokines decreased from baseline to three months, although significance was not reached. This trend may indicate that eyelid warming reduces the inflammatory load on the ocular surface, which could be explained by improved meibomian gland function and hence the quality of the tear film lipid layer. The lipid layer prevents tear film evaporation, and subsequent cell damage and inflammation at the ocular surface [13]. Contrasting the findings from baseline to three months, most of the cytokine concentrations seemed to increase from baseline to six months. This difference could possibly be explained by decreasing compliance to treatment throughout the study period. Alternatively, eyelid warming could impact the cytokine concentrations differently throughout the period of treatment. Future studies should be designed to improve understanding of how treatment affects the cytokine profile in MGD and DED.

Inflammation at the ocular surface is partly responsible for damage to the corneal epithelium observed in DED [6]. Such damage could be visualized as OSS [22]. We found several correlations between changes in cytokine concentrations and changes in the OSS score from baseline to three months. These findings could imply that the cytokine levels influence the degree of corneal and conjunctival epithelial damage in MGD patients treated with eyelid warming. On the other hand, changes in the extent of epithelial damage may also have impacted the cytokine concentrations during treatment. Our study failed to prove significant alterations in specific cytokines. It could therefore be suggested that eyelid warming is insufficient to improve damage to the ocular surface epithelium and hence reduce amount of inflammatory tear cytokines. Nevertheless, our findings signify a close connection between the level of tear cytokines and OSS due to MGD and DED. The results also indicate that OSS may predict the treatment effect on ocular surface inflammation. This further highlights the importance of OSS in the evaluation of treatment responses in MGD patients.

Significant correlations were found between changes in OSS and six cytokines (GCP-2, GM-CSF, IL-8, IL-10, IL-16, and MIF). We reviewed the literature regarding these six cytokines and their relation to DED. The level of GM-CSF was not increased in tear fluid from patients with mild to moderate DED compared to healthy controls [23]. The classification of DED was based on the Ocular Surface Disease Index questionnaire. Furthermore, Yucekul et al. did not find differences in the tear concentration of GM-CSF between subjects with MGD and healthy individuals [24]. However, GM-CSF was significantly upregulated at the ocular surface in a murine model of DED [25]. GM-CSF contributed to the pathogenesis of DED by promoting activation and migration of macrophages. A systematic review (meta-analysis) revealed that DED patients had higher levels of IL-8 and IL-10 in the tear fluid compared to controls [26]. Higher levels of IL-8 were also found in patients with MGD. Increased concentrations of IL-8 may recruit T lymphocytes to the ocular surface and cause tissue damage, and symptoms and signs of DED [4]. Accordingly, higher levels of IL-8 have been connected to more pronounced OSS [20]. Na and coworkers showed that the level of IL-16 was significantly increased in tear fluid from patients with DED [27]. Their results also suggested that higher concentrations of IL-16 were associated with more severe DED. The expression of MIF in the lacrimal gland was elevated in a mouse model of keratoconjunctivitis sicca [28]. In this study, botulinum toxin type B was injected into the lacrimal gland to induce dry eye. No publications were found concerning DED and GCP-2.

Reduced TBUT could be a sign of impaired function of the tear film lipid layer due to MGD [9]. In our study, altered TBUT was associated with alteration in the level of MCP-3. Consequently, a change in the lipid layer function or stability may have been followed by a change in the concentration of this cytokine. This correlation could possibly be connected to reduced inflammation in the eyelids and MGs, and hence improved lipid layer quality, following eyelid warming. It could also be related to the role of the lipid layer in preventing tear film evaporation, cell damage, and inflammation at the ocular surface. These mechanisms are consistent with the negative correlation between TBUT and MCP-3 found in the current study, which means that longer TBUT is related to lower levels of MCP-3.

The results from the whole group of patients were emphasized in this study. This was in accordance with its aim of investigating changes in the tear cytokine profile after eyelid warming in subjects with MGD. Moreover, merging the two intervention groups gave more power to investigate the hypothesis. However, the two treatments could have influenced the ocular surface inflammation and the cytokine composition differently, which may have given higher variability in the results. Despite this possible variability, significant correlations were revealed, which suggests that the association between changes in OSS and cytokines levels are independent of a specific therapy. Compliance to treatment dropped during follow-up, mainly from three to six months. Only three months of treatment may have been insufficient to alter the inflammatory condition at the ocular surface and change the tear cytokine levels significantly. Furthermore, a high number of cytokines were analyzed in our study. As a result, the statistical analysis had to take into account the multiple testing. The study was therefore less suitable to detect changes in specific cytokines. Lack of blinding of examiners was another limitation of this study. Finally, exclusion of patients requiring anti-inflammatory treatment has omitted individuals with more severe inflammation.

## 5. Conclusions

In conclusion, our study implies a close connection between the level of tear cytokines and OSS in MGD patients treated with eyelid warming. However, the study was unable to detect any significant changes in cytokine concentrations after treatment. These findings suggest that eyelid warming is insufficient to alter tear cytokine levels in MGD significantly in mild to moderate cases of the disease in this study. Future studies should compare the impact of substantially different treatments on the tear cytokine levels and clinical parameters in patients with MGD of various severities.

## Figures and Tables

**Table 1:** Overview of cytokines included in the analysis.

Name	Abbreviation	Chemokine name
6Ckine	—	CCL21
B-cell-attracting chemokine 1	BCA-1	CXCL13
Cutaneous T-cell-attracting chemokine	CTACK	CCL27
Eosinophil chemotactic protein 1	Eotaxin-1	CCL11
Eosinophil chemotactic protein 2	Eotaxin-2	CCL24
Eosinophil chemotactic protein 3	Eotaxin-3	CCL26
Epithelial-derived neutrophil-activating peptide 78	ENA-78	CXCL5
Fractalkine	—	CX3CL1
Granulocyte chemotactic protein 2	GCP-2	CXCL6
Granulocyte macrophage colony-stimulating factor	GM-CSF	—
Growth-regulated oncogene-alpha	Gro-*α*	CXCL1
Growth-regulated oncogene-beta	Gro-*β*	CXCL2
I-309	—	CCL1
Interferon gamma	IFN-*γ*	—
Interferon gamma-induced protein 10	IP-10	CXCL10
Interferon-inducible T-cell-alpha chemoattractant	I-TAC	CXCL11
Interleukin 1*β*	IL-1*β*	—
Interleukin 2	IL-2	—
Interleukin 4	IL-4	—
Interleukin 6	IL-6	—
Interleukin 8	IL-8	CXCL8
Interleukin 10	IL-10	—
Interleukin 16	IL-16	—
Macrophage-derived chemokine	MDC	CCL22
Macrophage inflammatory protein 1*α*	MIP-1*α*	CCL3
Macrophage inflammatory protein 1*δ*	MIP-1*δ*	CCL15
Macrophage inflammatory protein 3*α*	MIP-3*α*	CCL20
Macrophage inflammatory protein 3*β*	MIP-3*β*	CCL19
Macrophage migration inhibitory factor	MIF	—
Monocyte chemoattractant protein 1	MCP-1	CCL2
Monocyte chemoattractant protein 2	MCP-2	CCL8
Monocyte chemoattractant protein 3	MCP-3	CCL7
Monocyte chemoattractant protein 4	MCP-4	CCL13
Monokine induced by interferon gamma	MIG	CXCL9
Myeloid progenitor inhibitory factor 1	MPIF-1	CCL23
Small inducible cytokine subfamily B member 16	SCYB16	CXCL16
Stromal cell-derived factor 1	SDF1	CXCL12
Thymus- and activation-regulated chemokine	TARC	CCL17
Thymus-expressed chemokine	TECK	CCL25
Tumor necrosis factor alpha	TNF	—

**Table 2:** Results from correlation analysis between changes in tear cytokine levels in the whole treatment group from baseline to three months and changes in ocular surface staining and tear film break-up time in the same period.

Clinical parameters	Cytokines	Mean effect	Standard error	*P* value
Ocular surface staining				
	GCP-2	2.36	0.92	0.042
	GM-CSF	−2.98	0.99	0.024
	IL-8	−1.35	0.46	0.026
	IL-10	1.04	0.41	0.045
	IL-16	1.36	0.50	0.035
	MIF	−2.44	0.89	0.033

Tear film break-up time				
	MCP-3	−17.78	6.71	0.038

Only statistically significant results (*p* < 0.05) are displayed.

## Data Availability

The raw data used to support the findings of this study have not been made available because anonymization was not possible.

## Supplementary Materials

Supplementary Materials CONSORT checklist for randomized controlled trials.
